# 

**Published:** 1882-12

**Authors:** 


### PROSPECTUS

**For Vol. III, 1882, of**

### The Independent Practitioner:

A MONTHLY JOURNAL,

DEVOTED TO

Medicine, Surgery, Obstetrics, Dentistry, Hygiene and Popular Science.

*This Journal is cosmopolitan in its circulation, management and scientific resources, and of equal interest to the specialist and general practitioner throughout the civilized world.*

*Its corps of editors and collaborators will represent Medicine, Surgery, Obstetrics, Dentistry, Hygiene and Popular Science, and in these we will seek for that which is new and useful, and give the refined findings to our readers. Politics, religious creeds and isms, or preferences in colleges and cliques will be excluded, and we will uphold truth and justice with independence.*

### Seven Reasons why every one interested in its Topics should subscribe now.

*1st. It contains original papers from the best men in the profession.*

*2d. Its translations and selections contain more valuable and practical matter in an equal space than any other Journal published.*

*3d. Its Book Reviews are thorough criticisms made by competent writers, and in the interest of the profession.*

*4th. It treats Dentistry as a specialty of general Medicine and Surgery, and endeavors to strengthen the relationship between the different branches of Medical and Surgical Science and Art.*

*5th. It furnishes original and selected matter of a high scientific character in its Popular Science Department.*

*6th. It gives notes and items of interest, relating to medical and scientific progress throughout the world.*

*7th.* **Each number contains 72 pages of reading matter,** *printed on fine white super-calendered paper, with new type; compares favorably with any Medical Journal published, and its subscription price is within the reach of all progressive practitioners.*

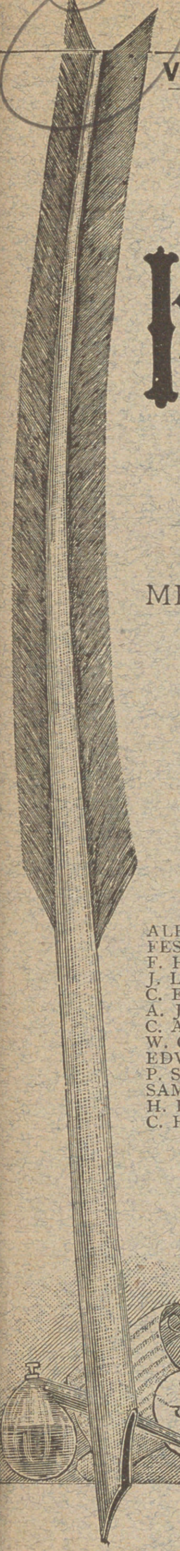

Vol. III. DECEMBER. 1882. No. 12.

S.G.O. INDEXED

THE

### Independent Practitioner:

A MONTHLY JOURNAL,

DEVOTED TO

MEDICINE, SURGERY, OBSTETRICS, DENTISTRY, HYGIENE, AND POPULAR SCIENCE.

**EDITORIAL CORPS:**

BASIL M. WILKERSON, D.D.S., M.D., EDITOR.

**Associates:**

——— M.D. ——— D.D.S.

**COLLABORATORS:**

ALF. L. LOOMIS, M.D.......... New York.
FESSENDEN N. OTIS, M.D...... "
F. H. BOSWORTH, M.D......... "
J. L. LITTLE, M.D............ "
C. E. FRANCIS, D.D.S., M.D.S.... "
A. J. C. SKENE, M.D........Brooklyn, N.Y.
C. A. MARVIN, D.D.S........ " "
W. C. BARRETT, M.D., D.D.S., Buffalo, "
EDWIN T. DARBY, M.D., D.D.S....Phil., Pa.
P. S. WALES, M.D., Surg.-Gen'l U. S. Navy.
SAMUEL C. BUSEY, M.D...Washington, D.C.
H. F. CAMPBELL, M.D.........Augusta, Ga.
C. H. MASTIN, M.D., LL.D......Mobile, Ala.

J. J. CHISOLM, M.D..........Baltimore, Md.
C. F. BEVAN, M.D............ " "
I. E. ATKINSON, M.D........ " "
H. McGUIRE, M.D...........Richmond, Va.
F. P. PORCHER, M.D........Charleston, S. C.
J. W. UNDERHILL, M.D.......Cincinnati, O.
JAS. T. WHITTAKER, M.D.... " "
J. RANSOHOFF, M.D., F.R.C.S. " "
F. FORCHHEIMER, M.D....... " "
A. R. JACKSON, M.D............Chicago, Ill.
S. V. CLEVENGER, M.D.......... " "
E. F. INGALS, M.D............ " "
J. H. CARSTENS, M.D.........Detroit, Mich.

NEW YORK:

*THE INDEPENDENT PRACTITIONER CO.,*

VANDERBILT BUILDING,

132 NASSAU STREET.

**$3.00 Per Annum, in Advance. - - - Single Copies, 30 Cents.**

*Entered at the Post-Office, New York City, as Second-Class matter.*

### PROSPECTUS, 1882.

THE INDEPENDENT PRACTITIONER is cosmopolitan in its scientific researches in Medicine, Surgery, Obstetrics, Dentistry, Hygiene, and Popular Science, and gives the refined findings to its readers. It is *independent* of colleges, cliques, isms, and any advertising firm, and fearlessly renders justice to all. Politics and religious creeds are excluded.

